# Transcriptome sequencing of the human pathogen *Corynebacterium diphtheriae* NCTC 13129 provides detailed insights into its transcriptional landscape and into DtxR-mediated transcriptional regulation

**DOI:** 10.1186/s12864-018-4481-8

**Published:** 2018-01-25

**Authors:** Manuel Wittchen, Tobias Busche, Andrew H. Gaspar, Ju Huck Lee, Hung Ton-That, Jörn Kalinowski, Andreas Tauch

**Affiliations:** 1 Center for Biotechnology (CeBiTec), Bielefeld University, Bielefeld, Germany; 2 Institute for Biology-Microbiology, Freie Universität Berlin, D-14195 Berlin, Germany; 3 Department of Molecular Biology and Biophysics, University of Connecticut Health Center, Farmington, CT USA; 4 Department of Microbiology & Molecular Genetics, University of Texas McGovern Medical School at Houston, Houston, USA; 5 Present address: Biological Resource Center, Korea Research Institute of Bioscience and Biotechnology, 181 Ipsin-gil, Jeollabuk-do, 56212 Republic of Korea

**Keywords:** *Corynebacterium diphtheriae*, Transcriptome sequencing, RNA-Seq, Transcription start site, Promoter, DtxR, Diphtheria toxin

## Abstract

**Background:**

The human pathogen *Corynebacterium diphtheriae* is the causative agent of diphtheria. In the 1990s a large diphtheria outbreak in Eastern Europe was caused by the strain *C. diphtheriae* NCTC 13129. Although the genome was sequenced more than a decade ago, not much is known about its transcriptome. Our aim was to use transcriptome sequencing (RNA-Seq) to close this knowledge gap and gain insights into the transcriptional landscape of a *C. diphtheriae tox*^+^ strain.

**Results:**

We applied two different RNA-Seq techniques, one to retrieve 5′-ends of primary transcripts and the other to characterize the whole transcriptional landscape in order to gain insights into various features of the *C. diphtheriae* NCTC 13129 transcriptome. By examining the data we identified 1656 transcription start sites (TSS), of which 1202 were assigned to genes and 454 to putative novel transcripts. By using the TSS data promoter regions recognized by the housekeeping sigma factor σ^A^ and its motifs were analyzed in detail, revealing a well conserved −10 but an only weakly conserved −35 motif, respectively. Furthermore, with the TSS data 5’-UTR lengths were explored. The observed 5’-UTRs range from zero length (leaderless transcripts), which make up 20% of all genes, up to over 450 nt long leaders, which may harbor regulatory functions. The *C. diphtheriae* transcriptome consists of 471 operons which are further divided into 167 sub-operon structures. In a differential expression analysis approach, we discovered that genetic disruption of the iron-sensing transcription regulator DtxR, which controls expression of diphtheria toxin (DT), causes a strong influence on general gene expression. Nearly 15% of the genome is differentially transcribed, indicating that DtxR might have other regulatory functions in addition to regulation of iron metabolism and DT. Furthermore, our findings shed light on the transcriptional landscape of the DT encoding gene *tox* and present evidence for two *tox* antisense RNAs, which point to a new way of transcriptional regulation of toxin production.

**Conclusions:**

This study presents extensive insights into the transcriptome of *C. diphtheriae* and provides a basis for future studies regarding gene characterization, transcriptional regulatory networks, and regulation of the *tox* gene in particular.

**Electronic supplementary material:**

The online version of this article (10.1186/s12864-018-4481-8) contains supplementary material, which is available to authorized users.

## Background

*Corynebacterium diphtheriae* is a Gram-positive bacterium causing the communicable disease diphtheria in humans by colonizing the upper respiratory tract or skin. Although a vaccine was introduced more than 100 years ago by von Behring, outbreaks still occur worldwide. A clinical isolate from a severe diphtheria outbreak in Eastern Europe in the 1990s, named *C. diphtheriae* NCTC 13129, was subjected to genomic sequencing in 2003 [1]. The genome of this *tox*^+^ strain has a size of about 2.5 Mbp with a G + C content of about 53% [1]. The published genomic information is a sound basis for further studies particularly concerning the pathogenicity of this bacterium [2–5].

Considered as the most important virulence factor of *C. diphtheriae*, diphtheria toxin (DT), encoded by the corynephage *tox* gene, has been studied extensively, with its structure [6–9] and mode of action [10, 11] now well characterized. Strain NCTC 13129 also harbors three pilus gene clusters, which encode three distinct adhesive pilus types that are assembled by sortase enzymes and critical for bacterial virulence [12, 13]. These pilus gene clusters and their variations are also identified in many pathogenic isolates from cases of diphtheria, endocarditis and pneumonia [3]. It is important to note here that *C. diphtheriae* mutants devoid of pili or DT are highly attenuated in the *Caenorhabditis elegans* and rodent models of infection [14, 15], supporting that DT and pili are the major virulence factors in *C. diphtheriae*.

Quite early the effect of increased DT is produced when *C. diphtheriae* is grown under iron-limiting conditions, and the basis of this modulation has been well studied and attributed to the transcriptional regulator DtxR [16–18]. In the presence of iron, DtxR is activated, forming a dimer that binds a 19 bp-palindromic sequence within the *tox* promoter, hence repressing the expression of the *tox* gene; in the iron-limiting conditions, DtxR is deactivated, leading to expression of DT [18]. Further research showed that DtxR is a dual regulator of iron homeostasis in many bacteria and its binding site was found upstream of several iron uptake related genes, like siderophores and heme oxygenases [3, 19–23]. It is not clear, however, if DtxR regulates genes coding for pili and the sortase machines.

Although the regulator and the genetic origin of the *tox* gene was identified many years ago, only a few studies focus on other genetic properties (e.g. promoter) of the DT encoding gene [24, 25]. In all only a few studies regarding the transcriptional organization of *C. diphtheriae* were performed [19, 22, 26, 27]. Promoters and operon structures are not known for the majority of genes.

For the analysis of transcriptomes a broad range of techniques exists. Northern blotting [28], Reverse transcription PCR [29], RACE (Rapid Amplification of cDNA Ends) [30] or Microarrays [31] are suitable for the analysis of transcripts and / or transcript abundance. The major drawback of most of these techniques is the fact that they only allow the analysis of a few to several targets in parallel, rendering the analysis of whole transcriptomes difficult and time consuming. Microarrays are designed for high-throughput screening of transcripts and their abundances but give little information about the transcript’s sequence [32]. Transcriptome sequencing (RNA-Seq) solves a lot of these problems and delivers some unique features such as de novo gene discovery. The technique provides both, the characterization of transcripts with single nucleotide resolution and transcript quantification with a high dynamic range. It is therefore considered ideal for the analysis of complete transcriptomes [33]. RNA-Seq revealed an unexpected complexity of bacterial transcriptomes and shed light on novel transcripts like small and antisense RNAs [34, 35]. Furthermore, a deep view into the transcriptome can be used to improve genomic annotations by identifying novel transcripts and correcting translation start sites (TLS) [36, 37]. Next to the regular sequencing of full length mRNAs (also called whole transcriptome sequencing), which is used for transcription profiling and differential gene expression analysis [33, 34], new RNA-Seq protocols emerged, which allow the analysis of very specific RNA features. Specific RNA-Seq protocols were invented to exactly map transcript ends for the identification of transcription start sites (TSS) [38] or terminator structures [39], by keeping the benefits of high dynamic range and resolution. This data can be used to identify promoter regions, analyze 5′ or 3′ untranslated regions (UTRs) and their inherent regulatory elements such as riboswitches [36, 40, 41].

Another feature of RNA-Seq is the ability to identify operon structures [38, 39]. Operon detection based on genomic data relies on function prediction of genes, the proximity of genes to each other and the encoding strand [42, 43]. The disadvantage of this approach is the requirement of a good genome annotation, since unknown genes cannot be assigned to operons. In addition, the combination of whole transcriptome and TSS data enables the identification of sub-operons, which are shorter transcripts originating from the same primary operon with an internal TSS [38, 39, 41].

This study aims to use RNA-Seq to gain insights into the transcription start sites (TSS) by enrichment of native 5′-ends of RNA and the transcriptional profile of *C. diphtheriae* wild type and Δ*dtxR* mutant strains by using whole transcriptome libraries. The obtained TSS data was further analyzed to get information about promoters and 5’-UTRs, the shorter of which only contain ribosomal binding sites and the longer ones may contain complex regulatory structure such as riboswitches, RNA thermometers or attenuators. Furthermore the wild type whole transcriptome data was used to detect operon structures. By combining the primary 5′-end and the whole transcriptome data of the wild type possible sub-operon structures can be characterized.

The whole transcriptome of the *C. diphtheriae* wild type and the Δ*dtxR* mutant was sequenced and compared to identify differentially expressed genes. The positions of DtxR binding sites relative to detected TSS are investigated. As the DT encoding gene *tox* is essential for the pathogenicity of *C. diphtheriae* we give detailed insights on the transcriptional landscape of this important gene. To the best of the authors’ knowledge the data presented here is the first RNA-Seq analysis of *C. diphtheriae*.

## Methods

### Bacterial strains and culture conditions

*C. diphtheriae* strains were grown in heart infusion broth (HIB) or on heart infusion agar (HIA), whereas *Escherichia coli* strains were grown on Luria broth (LB).

In-frame deletion of the *C. diphtheriae* *dtxR* gene was performed according to a published protocol [12]. Briefly, primer sets dtxR-A/B and dtxR-C/D were used to amplify 600 bp fragments upstream and downstream of *dtxR*, respectively, from the chromosomal DNA of strain NCTC 13129. The products were used for cross-over PCR with primers A and D to generate a 1.2 kbp fragment, while appending BamHI sites. The 1.2 kbp product was cloned into the BamHI sites of the conjugative vector pK18*mobsacB* [44]. DNA sequencing was employed to verify the insertion and the resulting plasmid was introduced into *E. coli* S17–1. The *E. coli* S17–1 strain harboring the resulting conjugative vector was used for deletion of *dtxR* in the parental strain NCTC 13129 via homologous recombination as previously described [12]. Confirmation of the defined *dtxR* deletion in the generated mutant was performed by PCR using the primers A and D (Additional file 1: Table S1).

### Cell sampling and RNA isolation

Overnight cultures of *C. diphtheriae* NCTC 13129 and its isogenic Δ*dtxR* mutant were used to inoculate fresh cultures in HIB at 37 °C. Total RNA was isolated from cells grown at exponential phase (OD_600_ = 0.5) using Trizol reagent (Invitrogen). The bacterial pellet obtained from 4 mL culture was resuspended in 1 mL Trizol, transferred into FastPrep Lysis Beads & Tube (MP Biomedicals) and mechanically lysed using beadbeater at a maximum speed for 20 s six times. After adding 200 μL chloroform to the lysed cells followed by centrifugation at 12,000×g for 15 min at 4 °C, the aqueous supernatant was taken and then precipitated using 500 μL isopropanol. Afterwards, crude RNA samples were treated with DNase I (Roche Diagnostics). After purification using phenol/chloroform/isoamyl alcohol (ratio 25:24:1), RNA was precipitated with ethanol. Purified total RNA pellets were dissolved in 50 μL RNase-free water. The purified RNA was quantified with a NanoDrop (Peqlab) and by Agilent RNA Nano 6000 kit on Agilent 2100 Bioanalyzer (Agilent Technologies). PCR was performed to assure no DNA remained in the samples.

### cDNA library preparations and sequencing

For whole transcriptome cDNA library preparations 2 μg total RNA from *C. diphtheriae* NCTC 13129 and the isogenic Δ*dtxR* mutant were used. Stable RNAs were depleted with the Ribo-Zero rRNA Removal Kit (Bacteria) according to manufacturer’s instructions (Epicentre). Afterwards the remaining mRNA was purified using RNA MinElute columns (Qiagen) and checked for quality with the Agilent RNA Pico 6000 kit and the Agilent 2100 Bioanalyzer (Agilent Technologies). Fragmentation of mRNA, reverse transcription to cDNA, adenylation of 3′-ends, adapter ligation and PCR amplification were performed according to TrueSeq Stranded mRNA library instructions (Illumina). Prior to paired-end sequencing of the cDNA libraries on an Illumina MiSeq, their quality and concentration were checked using the Agilent High Sensitivity DNA kit and the Agilent 2100 Bioanalyzer (Agilent Technologies).

For the primary 5′-end cDNA library 2× 5 μg RNA from the wild type *C. diphtheriae* NCTC 13129 was used. The preparation protocol has been described previously in detail [39]. In the present study, the experimental work-flow was slightly modified at three steps. Non-primary transcripts were digested with terminator exonuclease (Epicenre) at 30 °C for 60 min and subsequently at 42 °C for 30 min. The 5′ adapter ligation was performed for 60 min at 30 °C with 1 μL 60 μM adapter (Additional file 1: Table S1). After cDNA amplification the two libraries were purified and size-selected by gel electrophoresis for fragment sizes between 100 and 1000 bp. The cutoff of 100 bp was chosen to reduce the presence of adapter dimers in the library. Due to the fact that the library preparation involves the use of two adapters, which together have a length of 66 nt, only transcripts smaller than 40 nt are not present in the final RNA-Seq data. Sequencing was performed in single-read mode with 75 nt read length for the 5′-enriched cDNA library on an Illumina MiSeq.

### Bioinformatics data analysis

#### Read mapping and visualization

The paired-end reads of the whole transcriptome cDNA libraries from the wild-type and the Δ*dtxR* mutant were trimmed for low quality bases from both ends and a sliding window trimming (removing bases when the average quality per base in a window of 4 nt decreases below 15) using trimmomatic v0.36 [45]. Reads which were trimmed to a length shorter than 39 nt were discarded. The remaining paired-end reads were mapped with bowtie2 v2.2.7 [46] to the *C. diphtheriae* NCTC 13129 genome (RefSeq NC_002935.1) with default settings for paired-end read mapping. The single-end reads of the primary 5′-end cDNA library were trimmed from the 3′ end only with trimmomatic. The remaining reads with a minimal length of 39 nt were mapped with bowtie2 using default settings for single-end read mapping. All mapped sequence data was converted from SAM to BAM format to decrease usage of disk space with SAMtools v1.3 [47] and visualized and inspected with ReadXplorer v.2.2 [48].

#### Identification of transcription start sites

To automatically detect transcription start sites (TSS), the TSS detection mode of the ‘Transcription Analyses’ function of ReadXplorer was applied on the primary 5′-end cDNA library data. After empirical testing and inspection of various parameter sets based on the automatic parameter estimation by ReadXplorer the criteria for the automatic detection of putative TSS were a minimal coverage increase of 100% with at least 28 read starts at a particular genomic position. These values were found to result in an appropriate signal to noise ratio after manual inspection of the predicted TSS. The resulting list of predicted TSS was manually checked for false-positives. A putative TSS was considered as false-positive if no clear accumulation of read starts was observed at the particular genomic position and the putative TSS was found inside an uneven gradient of accumulated read starts, as it was often the case for putative TSS detected within a highly transcribed coding region.

#### Identification of novel transcripts

The TSS data from the primary 5′-end cDNA library were used to identify novel transcripts. The predicted TSS were associated with an annotated gene if they are located up to 500 bp upstream of the respective coding region. All TSS not associated with a known gene were assigned as novel transcripts. To further characterize this class of TSS it was divided into three groups: (a) TSS which are located between two annotated genes are considered as intergenic TSS; (b) TSS which are positioned inside an annotated gene are denoted as intragenic TSS; and (c) TSS which are located on the opposite strand of an annotated gene were classified as antisense TSS. To find novel transcripts which encode putative proteins the transcript length was estimated by the coverage from the wild type whole transcriptome cDNA library. The covered genomic region was searched for open reading frames (ORFs) by using UGENE [49] with AUG, GUG, UUG and CTG as start codon settings. Additionally, the corresponding stop codon had to be located within the covered genomic region. The predicted ORFs were checked for potential homologous proteins using NCBI BLAST [50, 51]. In case no ORF or protein homologue was detected, the sequence downstream of the TSS was analyzed for potential ncRNAs and RNA motifs using RFAM [52]. Newly identified genes were assigned with a locus tag containing a unique identifier.

#### Analysis of sequence motifs

To find conserved DNA sequence motifs in the *C. diphtheriae* NCTC 13129 genome, the motif-finding software Improbizer [53] and the visualization program WebLogo 3 [54] were used. Depending on the assumed motif location different input sequences were used. For the identification of σ^A^ promoter motifs the DNA sequence 40 bp upstream of the TSS assigned to a gene were taken as input. Improbizer reported an extended −10 region consisting of four unpreserved leading bases, the conserved core hexamer and two unpreserved tailing bases. For simplification the identified region was truncated to the conserved core hexamer and used as −10 motif in this work.

As the −35 motif of σ^A^ promoters is more frequent in presence of a −10 motif with low similarity to consensus [55], only sequences upstream of non-optimal −10 regions were used to identify possible −35 motifs. In addition to that the maximal allowed distance between the −35 and the −10 motif was set to 23 bp.

For the determination of ribosomal binding sites all genes, including predicted proteins encoded by novel transcripts, with an 5’-UTR longer than 5 nt were considered and the DNA sequence 20 bp upstream of the translation start site (TLS) was used as input for Improbizer.

The identified motifs are represented in the text in upper case letters if the frequency for the particular base is > 80% and in lower case letters if the frequency is below 80% of all analyzed sequences.

#### Identification of operon structures

Two or more genes are transcriptionally connected and part of an operon if they are transcribed from a single promoter. The detection of operon structures in *C. diphtheriae* was performed with ReadXplorer [48]. For this purpose the read pairs (with a minimal mapping quality ≥30) from the wild type whole track cDNA libraries spanning two neighboring genes on the same strand were counted. If more than five spanning reads were found the two genes were assigned to an operon structure (primary operon). The process was continued consecutively for the next genes until no more genes could be assigned to that operon. All three wild type cDNA libraries from the replicates were combined for the operon detection to increase the number of reads in low coverage regions. Genes represented by novel transcripts encoding proteins or ncRNAs (i.e. tmRNA) were manually checked for operon structures, as ReadXplorer can only allocate already annotated genes to operon structures. The list of primary operons was further divided into two groups: experimentally validated operons which have an assigned TSS and predicted operons which do not have an assigned TSS to their first gene. Sub-operons were identified in case a TSS was detected for a posterior gene in a primary operon. All genes which could not be connected into an operon were considered as monocistronic transcripts.

#### Differential gene expression analysis

Prior to differential expression analysis the whole transcriptome data from the *C. diphtheriae* wild type and the Δ*dtxR* mutant cDNA libraries were processed as described under ‘Read mapping and visualization’. For differential expression analysis the reads belonging to genes of three replicates per condition were counted with ReadXplorer and tested for differential expression with DESeq2 [56] using default settings. Genes with a false discovery rate corrected *p*-value below 0.05 and a log_2_(fold change) above +1.0 or below -1.0, respectively, were considered to be differentially transcribed under the examined conditions.

## Results and discussion

### cDNA sequencing and mapping to the *C. diphtheriae* NCTC 13129 genome

Although the genome of *C. diphtheriae* NCTC 13129 was published in 2003 [1] only a few examples about the transcriptional organization, promoter motifs and non-coding RNAs are known [18, 19, 24, 27, 57]. To perform qualitative and quantitative analyses of the *C. diphtheriae* transcriptional landscape, a technology capable of single-nucleotide resolution with great accuracy is required. Furthermore a technology with high dynamic range is needed for the analysis of the quantitative transcriptome. The recent technology of cDNA sequencing or RNA-Sequencing (RNA-Seq) fulfills all requirements and allows qualitative and quantitative transcriptome analyses in parallel [34, 58, 59]. Therefore we constructed two different types of cDNA libraries: a primary 5′-end-specific cDNA library of the wild type and whole transcriptome cDNA libraries of the wild type and of an isogenic Δ*dtxR* mutant. Triplicates of the wild type strain and the Δ*dtxR* mutant were cultivated to exponential growth in heart infusion broth medium, resulting in six individual whole transcriptome strand-specific cDNA libraries. All libraries were sequenced using a strand-specific protocol and an Illumina MiSeq machine with a read length of 75 nt or 2 × 75 nt for single-end and paired-end reads, respectively. The reads were quality-trimmed with trimmomatic [45] and mapped with bowtie2 [46] to the *C. diphtheriae* NCTC 13129 genome, using default parameters. Between 96 to 99% of the reads were mapped to the genomic reference (Additional file 2: Table S2). For visualization and further analysis, the mapped reads were imported into ReadXplorer [48].

### Identification of transcription start sites of primary transcripts

The analysis of the primary 5′-end cDNA data with the software ReadXplorer [48] resulted in the automatic detection of 3987 putative transcription start sites (TSS) in the *C. diphtheriae* NCTC 13129 genome. After manual inspection of the automatically detected TSS, 2310 false-positive TSS and 21 TSS assigned to rRNA and tRNA were discarded, leaving a list of 1656 manually curated TSS. These TSS were divided into two main categories: TSS that can be associated with annotated genes of the reference genome and TSS that probably belong to novel, not yet annotated transcripts. TSS assigned to annotated genes were further split into two categories: genes with a single TSS (874 genes) and genes with multiple TSS (137 genes), the latter case containing a total of 328 TSS. The TSS belonging to novel transcripts were classified into three groups: (a) intergenic TSS where the novel transcript is located between two annotated genes, (b) intragenic TSS where the novel transcript is located in the annotated coding region and (c) antisense TSS where the novel transcript is located on the opposite strand of an annotated gene.

**Fig. 1:**
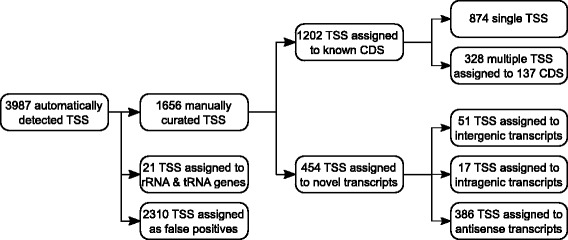
Classification of detected transcription start sites (TSS). The identification, curation and classification of TSS is shown. From the automatically detected 3987 TSS 2310 false positive TSS and 21 TSS belonging to rRNA and tRNA genes were removed, resulting in 1656 putative TSS assigned to different transcript types

All in all, 1202 TSS were assigned to protein-coding genes. In addition, 454 TSS associated with novel transcripts were detected which are not assigned to previously annotated genes: 51 TSS belong to novel intergenic transcripts, 17 TSS to intragenic and 386 TSS to antisense transcripts (Fig. 1; Additional file 4: Table S4 and Additional file 6: Table S6).

In rare cases a RNA might be rapidly dephosphorylated but somehow stabilized from degradation in the cell (for example by translating ribosomes or secondary structure). During library preparation this kind of RNA will be degraded leading to a loss of the TSS signal. For the 4.5S RNA and the 6C RNA no TSS could be detected, but both were abundant in the whole transcriptome data set. Nevertheless, the vast majority of all transcripts were covered.

### Identification of the house-keeping sigma factor σ^A^ promoter motif

**Fig. 2:**
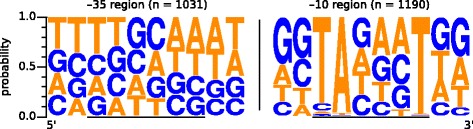
Promoter motifs for the sigma factor σ^A^ of *C. diphtheriae*. The size of the nucleotide represents the relative abundance of the particular nucleotide at this position. The −10 motif was found upstream of 1190 TSS and the −35 motif upstream of 1031 TSS. The data was visualized with Weblogo [54]

The identified TSS were used to analyze the upstream promoter regions for conserved motifs, representing DNA signals for the corynebacterial housekeeping sigma factor σ^A^. For this search the software Improbizer [53] was used to scan the upstream sequence of the detected TSS for conserved −10 motifs. The identification of the −35 motif was performed by searching the DNA sequence 23 bp upstream of non-optimal −10 motifs as the −35 motif of σ^A^ promoters is more frequent in presence of a −10 motif with low similarity to the consensus [55]. The −10 motif TAgaaT was identified upstream of 1190 (98.9%) TSS (Fig. 2). The recognized −35 motif (ttgcaa) is not well conserved, but it was found within a distance of 16–20 bp upstream of 1031 TSS that also possess a non-optimal −10 motif. The spacer length between the −10 motif and the detected TSS is 6 to 9 bp with a mean of 6.9 bp. The TSS itself is mainly (91%) a purine (A or G). The determined −10 motif and spacer length are in good agreement with data from *C. glutamicum* [39, 60, 61], a non-pathogenic relative of *C. diphtheriae*. The −35 motif of the *C. glutamicum* σ^A^ promoter (ttgcaa) is identical to that of *C. diphtheriae* and also not well conserved [39]. The comprehensive promoter data presented here lays the cornerstone for an in depth analysis of promoter motifs, which has already been done for *C. glutamicum* [55].

### Characteristics of 5′-untranslated regions (5’-UTRs)

#### 5’-UTR length distributions

**Fig. 3:**
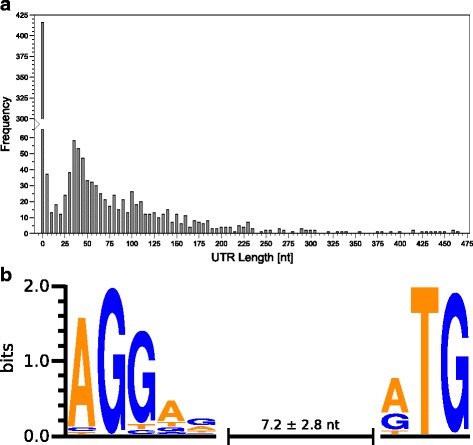
Histogram of 5’-UTR lengths and Ribosomal binding site motif in *C. diphtheriae*. **a** For the 5’-UTR analysis 1232 TSS from known genes and novel transcripts were used. The distribution of 5’-UTR lengths ranges from 0 nt (leaderless) to a maximum of 463 nt. Transcripts with a 5’-UTR of up to 5 nucleotides make up one third of all protein-coding genes. The bars represent UTR length increments of five nucleotides (1–5 nt, 6–10 nt, etc.), except for the first bar which represents UTR length of zero nucleotides. **b** Ribosomal binding site motif and mean distance to start codon. All 5’-UTRs longer than 5 nt were used for motif search. The y-axis shows the information content in bits for every nucleotide position. The diagram was created with Weblogo [54]

By analyzing the region between the TSS and the translation start site (TLS) in the primary 5′-end data, it was possible to obtain information on 5′-untranslated regions (5’-UTRs) in mRNA. The set of 1202 TSS assigned to known and 29 intergenic TSS assigned to novel protein-coding regions were used to characterize the 5’-UTRs. The length of the 5’-UTRs in *C. diphtheriae* mRNAs varies from 0 nt to 463 nt. The latter distance is for gene DIP1924A, a novel transcript identified in this study, encoding a hypothetical protein (Fig. 3a). Leaderless transcripts are mRNAs with 5’-UTRs that are too short for harboring a ribosomal binding site (RBS). Therefore, we categorized all genes with a 5’-UTR length from 0 to 5 nt as leaderless, as they cannot contain a canonical RBS with spacer. By using the primary 5′-end data 20% (452 of 2265) of the *C. diphtheriae* genes were found to be translated from leaderless transcripts (Additional file 3: Table S3). A large number of leaderless transcripts is a common feature of Actinobacteria [62] in general and Corynebacteria in particular, as the *C. glutamicum* transcriptome contains ~ 33% leaderless transcripts [39].

Further, the start codon usage was analyzed in both classes of transcripts, leaderless and leadered transcripts. For both classes AUG is the most frequently used start codon, followed by GUG. Around 80% of the leaderless transcripts contain an AUG, while only 62% of the leadered transcripts use this triplet as a start codon. The start codon UUG (~ 8%) is only found in transcripts having a ribosomal binding site.

As shown in Fig. 3a, a minor fraction (18%) of 5’-UTRs in *C. diphtheriae* has a length between 26 and 49 nt. These 5’-UTRs are long enough to contain a complete RBS with sufficient spacer length to the start codon, but are probably too short to harbor cis-regulatory elements.

#### Riboswitches and other RNA motifs

Another large group of 5’-UTRs have a length of > 100 nt. In total 264 (21%) genes are specified by a 5’-UTR of 100 nt or longer. These long 5’-UTRs might contain cis-regulatory elements which can influence transcription or translation of the mRNA by distinct sequence motifs or by folding into specific secondary structures. For various bacteria cis-regulatory elements in 5’-UTRs are known and can contain sequences of attenuators, riboswitches or binding sites for trans-regulatory elements [63, 64]. To find possible cis-regulatory elements in the 5’-UTRs, the genome sequence of *C. diphtheriae* NCTC 13129 was analyzed with the software Infernal [65] and the Rfam database [52] as search space. The results were compared with the 5’-UTR data from the primary 5′-end data set. Seven regulatory elements were predicted in the *C. diphtheriae* NCTC 13129 genome sequence, of which five elements were found to be transcribed at the applied conditions (Table 1). In addition to riboswitches the predicted RNA motifs common in actinobacteria and named *mraW* RNA and *msiK* RNA, presumably involved in peptidoglycan synthesis and in sugar import [66, 67], respectively, were detected as transcribed.

**Table 1:** Predicted cis-regulatory elements in the 5’-UTRs. The predictions were obtained by using Infernal 1.1.2 with the Rfam 12.1 database and compared with the primary 5′-end data set. The list is sorted by Bit Score in descending order. Abbreviation: n.a., not applicable

Infernal prediction with Rfam						RNA-Seq detection			
Name	ID	Start	End	Bit Score	Strand	Status	Start	End	Gene
Cobalamin riboswitch	RF00174	1,066,117	1,066,317	90.8	+	observed	1,066,117	1,066,317	DIP1084
Cobalamin riboswitch	RF00174	862,214	862,414	81.8	+	not observed^a^	n.a.	n.a.	n.a.
*cspA*	RF01766	285,506	285,923	64.8	+	observed	285,506	285,923	DIP0320 / *cspA*
*mraW*	RF01746	1,640,095	1,639,987	70.2	–	observed^b^	1,640,095	1,639,987	DIP1606 / *mraW*
TPP riboswitch	RF00059	924,778	924,890	60.8	+	observed^c^	924,778	924,890	DIP0953
TPP riboswitch	RF00059	922,838	922,969	60.0	+	observed^c^	922,838	922,969	DIP0951
*msiK*	RF01747	509,334	509,277	48.2	–	observed	509,334	509,277	DIP0539
TPP riboswitch	RF00059	27,456	27,566	57.6	+	not observed^a^	n.a.	n.a.	n.a.

^a^No TSS detected and insufficient read coverage in that area

^b^Motif of the *mraW* region is located upstream of the TSS of DIP1606 / *mraW*

^c^Last base of TPP riboswitch is located in the respective CDS

Leader peptides are small peptides encoded upstream of some amino acid biosynthesis operons. Their translation leads to a differential folding of the attenuator RNA depending on the intracellular availability of certain amino acids [68]. In *C. diphtheriae* NCTC 13129 three putative leader peptide genes were identified: the *trp*, *ilvB* and *leu* leader peptide genes. Upstream of the first gene of the operon for tryptophan (W) biosynthesis, *trpB1*, the *trp* leader peptide (MTNMNAHN*WWW*RA*) encoded at nucleotide positions 2,456,505–2,456,545 bp was found, but no TSS was identified for the leader peptide gene. The *ilvB* leader peptide (MNIIRL*VV*ITTRRLP*) is encoded upstream of *ilvB* at nucleotide positions 1,081,747–1,081,794 bp with a TSS at the leader start position rendering it a leaderless transcript. The *ilvB* gene is involved into the biosynthesis of the amino acids isoleucine (I) and valine (V). The *leu* leader peptide (MNRAN*LLLL*RRGGSQA*) is encoded at nucleotide positions 230,506–230,455 bp upstream of *leuA*. The *leuA* gene encodes the first step in leucine (L) biosynthesis. Neither for *leuA* nor for its leader peptide gene a TSS could be assigned due to weak transcription.

#### Ribosomal binding site motif

By using the 5’-UTR sequence information, a scan for ribosomal binding sites (RBS) was performed. Analysis of 779 5’-UTRs with a length larger than 5 nt by the software Improbizer [53] resulted in the conserved RBS motif AGGag in about 87% of all analyzed 5’-UTRs (Fig. 3b). The mean distance between the predicted RBS and the translation start site (TLS) of the coding region is 7.2 ± 2.8 nt. The identified RBS motif of *C. diphtheriae* is identical and the determined mean distance from RBS to TLS is very similar to that of *C. glutamicum* [39]. This was expected since the RBS-binding 3′-end of the 16S rRNA is identical in both organisms.

#### Re-annotation of coding sequences and detection of novel transcripts

By knowing the exact position of transcription start sites (TSS) of mRNA in the *C. diphtheriae* NCTC 13129 genome it is possible to verify, correct and re-annotate predicted coding sequences in the reference genome sequence. Furthermore, novel transcripts can be detected in the genome sequence and annotated. Accordingly two scenarios were anticipated; we corrected the translation start site (TLS) of coding sequences if the TSS is located downstream of the annotated TLS: a) In case the TSS is located at the first base of a potential start codon (ATG or GTG) that is in-frame with the annotated CDS, the TLS is shifted to the TSS position, resulting in a leaderless transcript. b) In case the TSS is not located at a start codon, the TLS is shifted to the next downstream in-frame start codon, resulting in a shortened CDS with a 5’-UTR of a length greater than 0 nt. By applying the two rules mentioned above, 120 TLS of predicted coding regions were corrected, of which 104 CDS are leaderless and 16 CDS have a 5’-UTR length greater than 5 nt (Additional file 3: Table S3 and Additional file 5: Table S5). These corrections were cross-checked by amino acid sequence similarity searches to orthologous proteins in databases and considered in the analysis of the 5’-UTR length distributions and in the motif searches.

By analyzing the intergenic, intragenic and antisense TSS, it is also possible to identify novel transcribed regions in the genome of *C. diphtheriae* NCTC 13129. As mentioned above, 454 TSS were classified as novel transcripts (Fig. 1). The intergenic TSS indicate novel not yet annotated coding regions or non-coding RNAs (ncRNAs). Only a few intergenic (51) and intragenic (17) TSS were detected. It is not clear which function these intragenic TSS have in *C. diphtheriae*, as they might lead to shortened proteins. For a range of organisms, e.g. bacteria [38], viruses [69] and eukaryotes [70], intragenic TSS and their shortened gene products have been described. These intragenic transcripts might contain regulatory regions which increase the genomic information content [71].

**Fig. 4:**
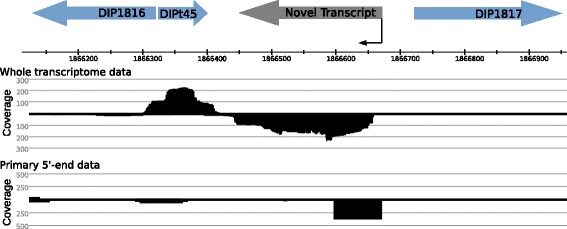
Illustration of an intergenic TSS assigned to a novel transcript. The intergenic TSS at position 1,866,670 bp of the *C. diphtheriae* NCTC 13129 genome has a corresponding coverage in the downstream direction, giving hint for a novel, not yet annotated transcript. An ORF given the locus tag DIP1817A was found, which is predicted to encode a helix-turn-helix (HTH) family transcriptional regulator

To find novel protein-coding transcripts, the sequences downstream of intergenic TSS were analyzed for open reading frames (ORFs) using the software UGENE [49]. In case a potential ORF was found, its amino acid sequence was analyzed with BLASTp [50, 51] to detect possible protein homologues in public databases. In case no ORF or protein homologue was found, the sequence downstream of the TSS was searched for ncRNAs or RNA motifs with RFAM [52]. For 29 of the 51 intergenic TSS a potential ORF was found and assigned with a distinct locus tag. The two ncRNAs tmRNA and RNase P M1 RNA were also identified as novel transcripts (Additional file 6: Table S6). Around 40% of the newly detected ORFs were predicted to encode hypothetical proteins or proteins of unknown functions, but some proteins with metabolic function were also predicted. These proteins encode formate C-acetyltransferase, an ammonium transporter, magnesium chelatase, and glycine dehydrogenase. In addition, a gene encoding a putative helix-turn-helix (HTH) family transcriptional regulator (DIP1817A) was identified in the intergenic region between DIP1816 and DIP1817 (Fig. 4).

#### Analysis of operon structures by combining the primary 5′-end and the whole transcriptome data sets

By combining the primary 5′-end and the whole transcriptome data sets it is possible to obtain further insights into the transcriptional landscape of *C. diphtheriae* NCTC 13129, in particular into operon structures. An operon is a polycistronic transcript consisting of at least two genes transcribed from a common promoter. We defined a requirement of at least five reads spanning two adjacent genes to assign them to a primary operon. A primary operon was considered as ‘experimentally validated’ if a TSS was assigned to the first gene of that operon and ‘experimentally validated by read pairs’ if no TSS could be detected. In case an additional TSS is located inside of a deduced primary operon, a shortened transcript is generated during gene expression that defines a sub-operon, containing one or more genes. All genes not assigned to an operon were classified as monocistronic transcripts and were categorized regarding their TSS detection.

**Fig. 5:**
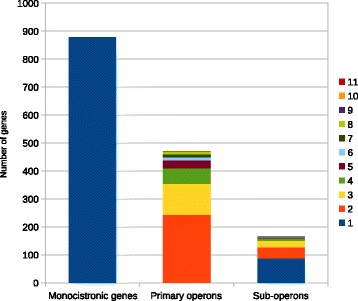
Number of monocistronic genes, primary operons and sub-operons of *C. diphtheriae* NCTC 13129. The number of genes included in primary and sub-operons is color-coded

Under the studied conditions 471 primary operons containing 1417 genes were deduced from the transcriptome data. Of the 471 primary operons 337 operons (72%) are experimentally validated, as a TSS was assigned to their first genes, leaving 134 operons as experimentally validated by read pairs only. When considering internal TSS, the primary operons contain 167 sub-operons (Fig. 5). The two ncRNAs tmRNA and RNase P M1 RNA are co-transcribed in operons with protein-coding regions. The tmRNA is part of an operon with the *smpB* (DIP0750) gene encoding the SsrA-binding protein. The M1 RNA is transcribed in a primary operon consisting of DIP1679, DIP1678, M1 RNA and DIP1677. This primary operon is further divided into two sub-operons ranging from DIP1678 to DIP1677 and from M1 RNA to DIP1677 indicating a complex expression pattern of this genomic region. For the 4.5S RNA (DIP0256) and the known actinobacterial 6C RNA no TSS was detected in this study.

The largest primary operon covers eleven genes which code for various ribosomal proteins of the 30S and 50S subunits. Ten operons covering eight genes exist in the *C. diphtheriae* transcriptome containing genes involved in various cellular functions from replication to carbohydrate metabolism (Table 2). A list of all detected operons and sub-operons of *C. diphtheriae* NCTC 13129 is provided in the Supplemental Material (Additional file 7: Table S7).

**Table 2:** Largest primary operons in *C. diphtheriae* NCTC 13129. The predictions from the eggNOG database (v4.5) were used to classify genes by their functions

Genes	Number of genes	Strand	TSS	Gene names and classification by eggNOG database
DIP0472 - DIP0482	11	+	detected	*rpsJ, rplC, rplD, rplW, rplB, rpsS, rplV. rpsC, rplP, rpmC, rpsQ*. Translation, ribosomal structure and biogenesis (ribosomal proteins)
DIP0203 - DIP0209	8	+	not detected	DIP0203, DIP0204, DIP0205, DIP0206, DIP0207, DIP0208, DIP0208A, DIP0209. Function unknown, phage proteins
DIP0407 - DIP0414	8	+	detected	*hemE, hemG, hemL*, DIP0410, DIP0411, DIP0412, DIP0413, DIP0414. Coenzyme metabolism; Carbohydrate metabolism and transport; Post-translational modification, protein turnover, and chaperones
DIP0438 - DIP0445	8	+	detected	DIP0438, DIP0439, DIP0440, DIP0441, DIP0442, DIP0443, DIP0444, DIP0445.Inorganic ion transport and metabolism; Function unknown
DIP0719 - DIP0726	8	+	detected	DIP0719, DIP0720, DIP0721, DIP0722, DIP0723, DIP0724, DIP0725, *uvrD*. Function unknown; Replication, recombination and repair; Inorganic ion transport and metabolism
DIP0969 - DIP0976	8	+	detected	DIP0969, DIP0970, DIP0971, DIP0972, *fdxA*, DIP0974, DIP0975, DIP0976. Signal transduction mechanisms; Amino Acid metabolism and transport; Function unknown; Energy production and conversion
DIP1248 - DIP1241	8	–	detected	DIP1248, DIP1247, DIP1246, DIP1245, DIP1244, DIP1243, *tatA*, *tatC*. Intracellular trafficking, secretion, and vesicular transport; Transcription; Amino Acid metabolism and transport; Post-translational modification, protein turnover, and chaperones; Function unknown
DIP1603 - DIP1596	8	–	not detected	*murE, murF, mraY, murD*, DIP1599, *murG, murC*, DIP1596. Cell wall, membrane, envelop biogenesis
DIP1720 - DIP1713	8	–	not detected	*dnaJ2*, DIP1719, DIP1718, DIP1717, DIP1716, DIP1715, *era*, *recO*. Replication, recombination and repair; Cell wall, membrane, envelop biogenesis; Coenzyme metabolism; Inorganic ion transport and metabolism; Function unknown; Signal transduction mechanisms; Post-translational modification, protein turnover, and chaperones
DIP1779 - DIP1772	8	–	not detected	*obgE*, DIP1778, *proB, proA, nadD*, DIP1774, DIP1773, DIP1772. Function unknown; Carbohydrate metabolism and transport; Coenzyme metabolism; Amino Acid metabolism and transport; Energy production and conversion
DIP1857 – DIP1850	8	–	detected	*clpS*, DIP1856, DIP1855, DIP1854, *murI*, DIP1852, *rph*, DIP1850. Nucleotide metabolism and transport; Translation, ribosomal structure and biogenesis; Function unknown; Cell wall, membrane, envelop biogenesis; Amino Acid metabolism and transport; Secondary metabolites biosynthesis, transport, and catabolism; Post-translational modification, protein turnover, and chaperones

The number of monocistrons in the *C. diphtheriae* genome accounts for 878 genes (38% of the predicted genes), of which 550 genes (63%) were associated with a TSS in this study (Fig. 5). Considering the number of genes assigned to primary operons as well as monocistrons with an assigned TSS, nearly 87% of all annotated genes of *C. diphtheriae* NCTC 13129 were detected as actively transcribed in this study.

We compared our results from the operon detection to the *in silico* operon predictions from the Database of prOkaryotic OpeRons (DOOR) [72]. Our RNA-Seq based operon detection is in agreement with the vast majority (89%) of all primary operons predicted by DOOR. The missing 11% were not evaluated in this study due to insufficient read coverage in the respective regions caused by low transcription.

Considering all detected TSS and identified primary operons as well as monocistrons, around 87% of all annotated genes are represented in this study. The remaining genes not covered are most likely due to two reasons: We analyzed transcription during exponential growth phase in complex media. Genes only active in other growth phases or conditions are not considered. Furthermore the applied method for capturing 5′-ends of transcripts relies on the fact that actively transcribed RNA is triphosphorylated at the 5′-end, which might not be the case for some transcripts. However, the large majority of transcripts was covered in this study.

#### Analysis of the DtxR regulon by comparing two whole transcriptome data sets

The diphtheria toxin repressor (DtxR) is the transcriptional regulator of iron homeostasis and the diphtheria toxin gene *tox* in *C. diphtheriae* and therefore important for the pathogenicity of this bacterium [73]. The iron-sensing transcription regulator DtxR binds to the DtxR motif on the DNA under iron excess conditions and thereby regulates the expression of genes coding for proteins involved in iron metabolism [3, 4]. It was shown experimentally in *C. glutamicum* that DtxR is a dual regulator which represses genes related to iron uptake but activates genes related to iron storage under iron excess conditions [23]. To analyze the dual characteristics of DtxR in *C. diphtheriae*, we compared the genome-wide transcription profile of a Δ*dtxR* mutant with that of the wild type strain. The mapped paired reads were counted with ReadXplorer [48] and the DESeq2 tool [56] was used to measure differential transcription (Additional file 9: Table S8). To assure that all three biological replicates are suitable for comparison, the normalized read counts calculated from DESeq2 were plotted against each other and the Pearson correlation coefficient R^2^ was calculated. All replicates from both strains showed high R^2^ values demonstrating the highly reproducible experimental set-up (Additional file 8: Figure S1).

**Fig. 6:**
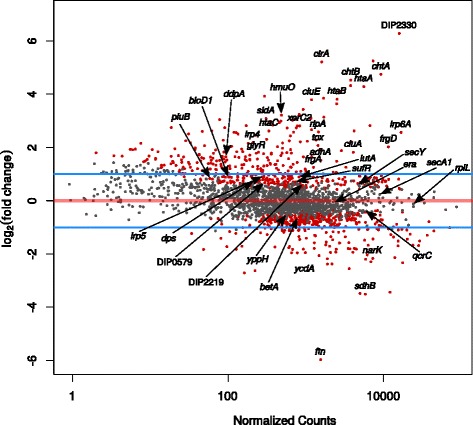
M/A plot of differentially transcribed genes in the Δ*dtxR* mutant. Genes with an adjusted *p*-value below 0.05 are shown in red. The blue lines indicate the log_2_(fold change) threshold of +1.0 and −1.0, respectively. Genes with a known or predicted DtxR binding site are labeled

Genes with a log_2_(fold change) (LFC) above +1.0 or below −1.0 and an adjusted *p*-value below 0.05 were considered as differentially transcribed. In total 224 genes showed elevated transcript levels and 113 genes decreased transcript levels in the mutant when compared with the wild type (Fig. 6 and Additional file 9: Table S8). The deletion of the dtxR gene had a remarkable influence on the transcriptome of the mutant strain affecting around 15% of all genes either directly or by indirect effects. The gene with the largest log_2_(fold change) (LFC 6.28) is DIP2330 encoding a putative secreted protein of unknown function. Among the 40 genes with known or predicted DtxR binding sites, 25 (63%) were differentially transcribed in the Δ*dtxR* mutant. According to the state of differential transcription the genes with DtxR binding sites are either repressed or activated by DtxR (Table 3). The majority of the 25 differentially transcribed genes showed an enhanced transcription in the mutant strain and are therefore repressed by DtxR in the wild type under iron excess conditions. Among this group of genes are those encoding hemin receptors, iron transporters and iron siderophores. The genes *ftn*, *sdhB*, *narK* and *ycdA* are weakly transcribed in the mutant. These genes are therefore probably activated by DtxR in the wild type in an iron-rich condition. The iron storage gene *ftn* showed the lowest transcription in the *dtxR* mutant when compared with the wild type, which might underline the dual regulatory function of DtxR. Intriguingly, *srtC*, coding for the pilus-specific sortase SrtC involved in the assembly of the SpaD-type pili [2], was stronger transcribed in the absence of *dtxR* (Additional file 9: Table S8; LFC 1.27). No other sortase and pilin genes were observed as differentially transcribed in the Δ*dtxR* mutant.

**Table 3:**
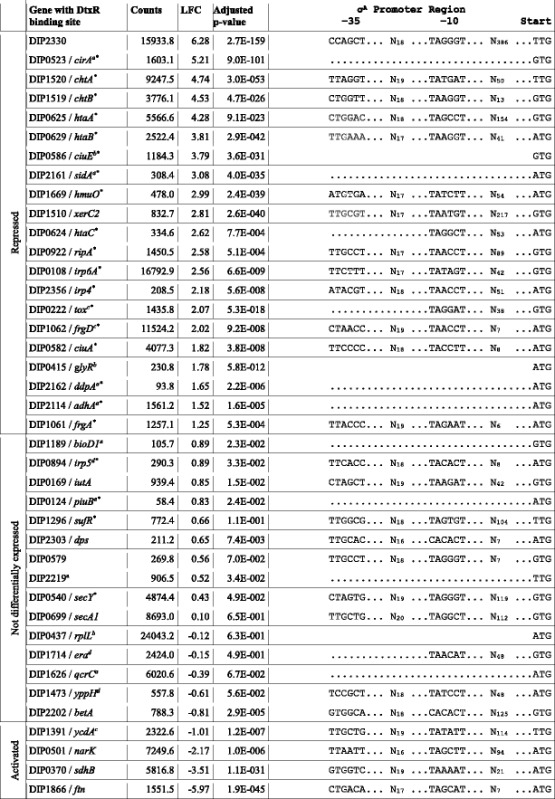
Differential expression and σ^A^ promoter region of genes with known or predicted DtxR binding site. Based on the differential expression values the genes are classified into three groups: repressed, activated and not differentially expressed. In case no TSS could be assigned to the gene only the translation start codon is shown. In case a TSS could be assigned, but no −10 motif could be identified the start codon is preceded with a dotted line. Counts, normalized read counts; LFC, log_2_(fold change); Start, start codon. The asterisks mark genes with an experimentally shown DtxR regulation

^a^No TSS detected, hence no promoter motif predictable

^b^Gene is part of a primary operon and lacks own TSS

^c^Multiple TSS detected and only TSS closest to DtxR binding site shown. In case of the *tox* gene the −10 motif of the TSS (TSS 2) closest to the start codon is shown

^d^Gene is the first one in a sub-operon and therefore has own TSS

The locations of binding sites and the respective TSS are in accordance with regulatory models. The DtxR binding sites of all DtxR-activated genes are located at least 37 bp upstream of the detected TSS. In contrast to that the DtxR binding sites of all DtxR-repressed genes overlap the −10 region of the σ^A^ promoter or are located downstream of the detected TSS. The mechanism of repression by DtxR most likely works by simply covering the promoter site and thereby preventing the RNA polymerase from binding or by roadblocking which forces the RNA polymerase to halt prematurely [74]. Binding of DtxR upstream of a promoter seems to have an activating effect on gene transcription but it is unclear how the mechanism of gene activation works. Nevertheless for some genes with known or predicted DtxR binding site no TSS was detected in this study presumably due to low transcription (Table 3).

#### Comprehensive transcriptomic view on the phage island and the *tox* gene encoding diphtheria toxin

*C. diphtheriae* NCTC 13129 is a *tox*^+^ strain as it carries a corynephage that harbors the diphtheriae toxin gene *tox*. The diphtheriae toxin (DT) is one of the strongest bacterial toxins and essential for the pathogenicity of *C. diphtheriae* [73, 75]. Here we use the primary 5′-end data and the whole transcriptome data to gain a comprehensive view on the transcriptional features of the phage island and the *tox* gene region in particular.

**Fig. 7:**
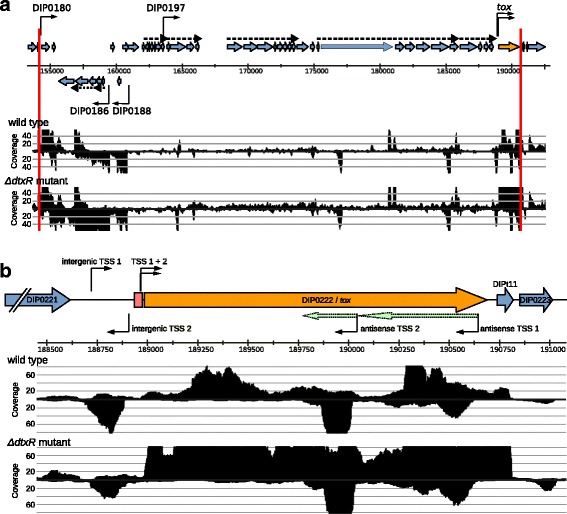
Transcription profile of the phage island and the *tox* gene. **a** The transcription profile of the phage island (framed by two red lines) of the wild type and the isogenic Δ*dtxR* mutant is shown. The genes which could be assigned with a TSS are labeled with a black arrow. Primary operon structures are indicated by dashed arrows. The *tox* gene is shown in dark yellow. TSS assigned to intergenic and antisense transcripts were omitted for clarity. **b** Detailed view on the monocistronic *tox* gene. It possesses two TSS (TSS 1 and TSS 2) with 5’-UTR lengths of 43 nt and 38 nt. The DtxR binding site (red box) is located close to the start codon of the CDS and overlaps both TSS and their corresponding −10 promoter motifs. Two additional TSS for putative antisense RNAs were found (light green arrows) and two additional TSS (intergenic TSS 1 and intergenic TSS 2) on the forward and reverse strand, respectively, were identified upstream of the *tox* gene, indicating two putative novel transcripts. The image is a modified screenshot from ReadXplorer [48] showing non-normalized coverage data of one exemplary replicate per condition

The phage island is located between the tRNA^Arg^ genes DIPt10 and DIPt11 and consists of 44 genes (from DIP0180 to DIP0222/*tox*). Only six TSS were assigned to corynephage genes. One of these genes is transcribed leaderless (DIP0180), while the others have varying 5’-UTR lengths ranging from 28 nt to 413 nt. Furthermore, six TSS assigned to putative antisense transcripts were detected. By operon analysis, the phage island is transcriptionally structured in 7 primary operons containing 34 genes and one sub-operon containing one gene (DIP0197), leaving the remaining 10 genes transcribed as monocistrons (Fig. 7a).

Interestingly, the transcription of genes in the middle of the phage island is relatively low compared to genes in the exterior regions of the island. This is the case for both wild type and Δ*dtxR* mutant strains. However, 20 genes (45%) in the phage island are differentially transcribed in the Δ*dtxR* mutant. These genes do not cluster in a specific region, as genes in the exteriors and the middle part of the phage island are affected. Although the *tox* gene is the only gene in the phage island with a DtxR binding site, additional 19 corynephage genes are stronger transcribed in the Δ*dtxR* mutant. Various genes encoding for phage repression and capsid assembly as well as the putative phage integrase and a putative transcription regulator are among these genes.

Although the *tox* gene is part of the corynephage genome, its expression is under bacterial control. Upstream of the *tox* gene a DtxR binding site is located that is blocked under high-iron conditions by the regulator protein DtxR encoded on the corynebacterial chromosome [16–18]. Many studies focus on the structure of the DT protein [6–8, 76] or its domains [9, 77–79] but only a few studies addressed the transcriptomic characteristics of the *tox* encoding gene [24, 25, 80].

In front of the *tox* coding region two TSS (named TSS 1 and TSS 2) with predicted σ^A^ promoter motifs were detected (Additional file 3: Table S3), resulting in 5’-UTRs of 43 nt and 38 nt respectively (Fig. 7b). The DtxR binding site is located 32 bp upstream of the start codon and overlaps the −10 promoter motif of both TSS. As expected, the transcription of *tox* was increased > 4-fold in the Δ*dtxR* mutant when compared with the wild type. In early studies of the *tox* transcription two overlapping promoters resulting in two TSS at positions −38 and −43 relative to the GTG start codon were found [25, 81]. As shown by site-directed mutagenesis the −10 motif proximal to the start codon is more active than the other [25]. Our primary 5′-end data supports these findings as the number of read starts at the TSS closer to the start codon (TSS 2) is ~ 5-fold higher compared to the distal TSS (TSS 1) (data not shown).

In addition to the TSS of the *tox* coding region, two putative antisense RNA (asRNA) and their TSS as well as two intergenic TSS were deduced from the transcriptome data (Fig. 7b). To the best of the authors’ knowledge this is the first description of *tox* related asRNA in *C. diphtheriae*. The two asRNA start close to the end of the *tox* CDS (asRNA TSS 1) and approximately in the middle of the CDS (asRNA TSS 2). The lengths estimated from overlapping reads are 580 nt for asRNA 1 and 260 nt for asRNA 2. Antisense RNAs have a broad range of functions effecting transcription, stability or translation of the sense mRNA [82]. However the function of the identified antisense RNAs is not known at this point.

The two intergenic TSS (intergenic TSS 1 and 2, Fig. 7b) indicate the presence of novel transcripts upstream of the *tox* gene. Both novel transcripts are on opposing strands facing each other. While the coverage of the transcript of the reverse strand is clearly visible, the coverage of the other transcript on the forward strand is very low. Both the BLAST search of potential ORFs and the ncRNA prediction by Infernal/RFAM gave any hints on possible functions. Although the *tox* gene regulation by DtxR is known for decades, our findings illustrate that the transcriptional landscape of this gene region is far more complex and still compelling. Further research is needed to shed light onto the complex transcriptional patterns in the *tox* gene region.

## Conclusion

This study comprises the first reported whole transcriptome and transcription start site (TSS) analysis of *C. diphtheriae* NCTC 13129. We provide a comprehensive list of TSS, promoter motifs and ribosomal binding sites as well as 5’-UTRs for the majority of genes. Furthermore, we corrected several predicted coding regions based on the experimentally detected TSS data and found hundreds of putative novel transcripts. By combining the whole transcriptome with the 5′-enriched cDNA library data operon and sub-operon structures were predicted. In addition, differential gene expression analysis of a *dtxR* deletion mutant was performed to identify the global effects of DtxR regulation that includes the diphtheria toxin gene *tox*. As the *tox* gene is a major factor contributing to the pathogenicity of *C. diphtheriae* we present a detailed analysis of the transcriptional landscape of this important gene region.

The findings presented here greatly expand the understanding of transcript regulation and provide a solid foundation for further transcriptome studies of this important pathogen. In particular the cornerstone was laid for in depth analyses of promoters motifs and for transcriptional analysis of host pathogen interactions. Furthermore, future studies might be focused on small and antisene RNAs, which could harbor new regulatory elements and functions.

## Additional files

Additional file 1: Table S1. Oligonucleotide sequences. (XLSX 5 kb)

Additional file 2: Table S2. Number of cDNA reads of the cDNA libraries. (XLSX 5 kb)

Additional file 3: Table S3. List of transcription start sites assigned to known CDS. (XLSX 70 kb)

Additional file 4: Table S4. List of intragenic and antisense TSS. (XLSX 15 kb)

Additional file 5: Table S5. List of corrected CDS start sites. (XLSX 9 kb)

Additional file 6: Table S6. List of intergenic TSS. (XLSX 8 kb)

Additional file 7: Table S7. List of operons, sub-operons and monocistrons. (XLSX 38 kb)

Additional file 8: Figure S1. Reproducibility of differential expression analysis with varying cDNA library replicates. (PDF 1397 kb)

Additional file 9: Table S8. List of differentially transcribed genes. (XLSX 418 kb)

## Abbreviations

5‘-UTR: 5‘-Untranslated region
DT: Diphtheria toxin
DtxR: Diphtheria toxin repressor
LFC: log_2_(fold change); a measure of change in gene transcription
RBS: Ribosomal binding site
RNA-Seq: RNA-Sequencing or transcriptome sequencing
TLS: Translation start site
TSS: Transcription start site
σ^A^: Sigma factor A
