# Supplementary material for: Transcriptome sequencing of the human pathogen Corynebacterium diphtheriae NCTC 13129 provides detailed insights into its transcriptional landscape and into DtxR-mediated transcriptional regulation
Source: BMC Genomics. 2018 Jan 25;19:82. doi: 10.1186/s12864-018-4481-8 (PMC5784534; doi:10.1186/s12864-018-4481-8)
Supplement: Supplementary file 8 — Reproducibility of differential expression analysis with varying cDNA library replicates. (PDF 1397 kb) [file 12864_2018_4481_MOESM8_ESM.pdf]

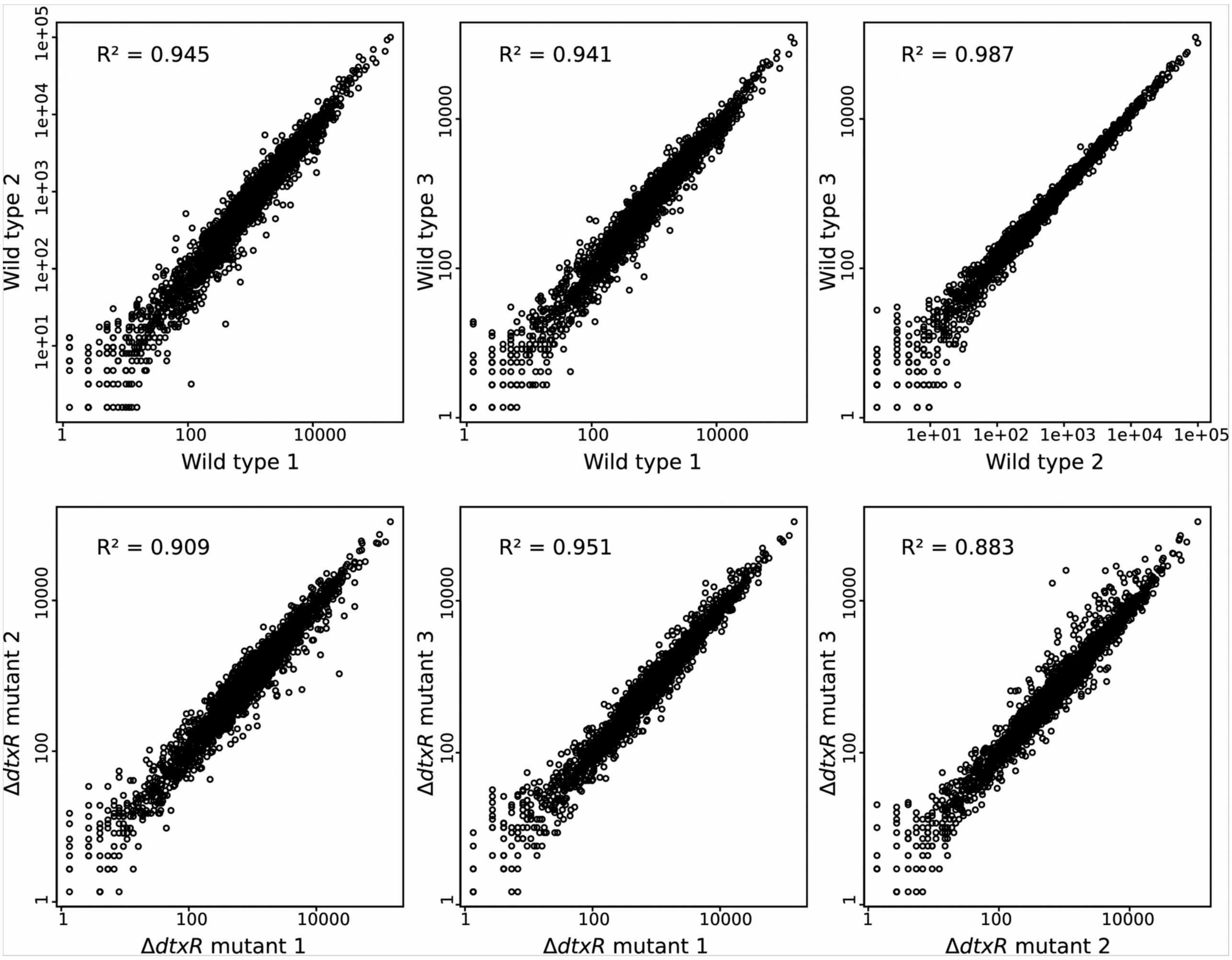

Additional File 8: Figure S1: Reproducibility of differential expression analysis with varying cDNA library replicates. For each pair of experimental replicates the normalized read counts for each gene were calculated with DESeq2 and plotted against each other. The Pearson correlation coefficient (R^2) is given as a measure of reproducibility.
